# Diisopropyl 1-(4-meth­oxy­phen­yl)-2,6-dimethyl-4-(3-nitro­phen­yl)-1,4-dihydro­pyridine-3,5-dicarboxyl­ate

**DOI:** 10.1107/S1600536811042073

**Published:** 2011-10-22

**Authors:** Kamini Kapoor, Vivek K. Gupta, Rajni Kant, Milind P. Pawar, Hitendra S. Joshi

**Affiliations:** a X-ray Crystallography Laboratory, Post-Graduate Department of Physics, University of Jammu, Jammu Tawi 180 006, India; b Chemistry Department, Saurashtra University, Rajkot 360 005, India

## Abstract

In the title compound, C_28_H_32_N_2_O_7_, the 1,4-dihydro­pyridine ring adopts a flattened boat conformation. The two benzene rings are approximately perpendicular to the dihydro­pyridine ring, forming dihedral angles of 84.29 (9) and 82.96 (9)° with the mean plane of the 1,4-dihydro­pyridine unit, whereas the ester groups are only slightly twisted relative to this plane, with dihedral angles of 10.6 (1) and 9.0 (1)°.

## Related literature

For background to the pharmaceutical applications of 1,4-dihydro­pyridine derivatives, see: Gaveriya *et al.* (2001 ▶); Shah *et al.* (2000 ▶, 2002 ▶); Marchalin *et al.* (2004 ▶); Chhillar *et al.* (2006 ▶).
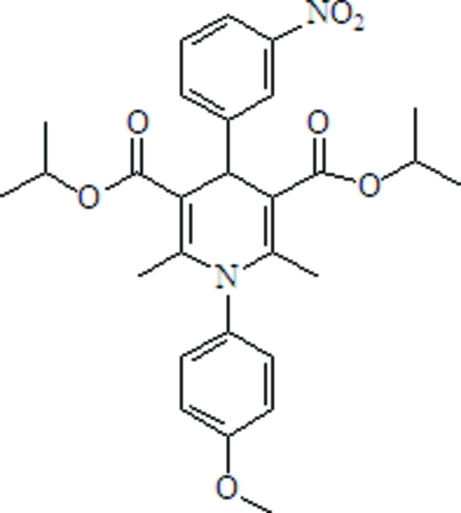


## Experimental

### 

#### Crystal data

C_28_H_32_N_2_O_7_
*M* *_r_* = 508.56
Triclinic, 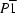

*a* = 9.5043 (8) Å
*b* = 10.7570 (7) Å
*c* = 15.1279 (12) Å
α = 90.501 (6)°
β = 105.873 (7)°
γ = 114.601 (7)°
*V* = 1339.27 (18) Å^3^
*Z* = 2
Mo *K*α radiation
μ = 0.09 mm^−1^
*T* = 293 K
0.30 × 0.20 × 0.20 mm

#### Data collection

Oxford Diffraction Xcalibur S diffractometer
Absorption correction: multi-scan (*CrysAlis RED*; Oxford Diffraction, 2007 ▶) *T*_min_ = 0.825, *T*_max_ = 1.000
8313 measured reflections
4688 independent reflections
2417 reflections with *I* > 2σ(*I*)
*R*_int_ = 0.043

#### Refinement

*R*[*F* ^2^ > 2σ(*F* ^2^)] = 0.069
*wR*(*F* ^2^) = 0.203
*S* = 0.93
4688 reflections
334 parameters
H-atom parameters constrained
Δρ_max_ = 0.23 e Å^−3^
Δρ_min_ = −0.23 e Å^−3^

### 

Data collection: *CrysAlis CCD* (Oxford Diffraction, 2007 ▶); cell refinement: *CrysAlis CCD*; data reduction: *CrysAlis RED* (Oxford Diffraction, 2007 ▶); program(s) used to solve structure: *SHELXS97* (Sheldrick, 2008 ▶); program(s) used to refine structure: *SHELXL97* (Sheldrick, 2008 ▶); molecular graphics: *ORTEP-3 for Windows* (Farrugia, 1997 ▶); software used to prepare material for publication: *PLATON* (Spek, 2009 ▶) and *PARST* (Nardelli, 1995 ▶).

## Supplementary Material

Crystal structure: contains datablock(s) I, global. DOI: 10.1107/S1600536811042073/gk2409sup1.cif

Structure factors: contains datablock(s) I. DOI: 10.1107/S1600536811042073/gk2409Isup2.hkl

Supplementary material file. DOI: 10.1107/S1600536811042073/gk2409Isup3.cml

Additional supplementary materials:  crystallographic information; 3D view; checkCIF report

## Figures and Tables

**Fig. 1.**
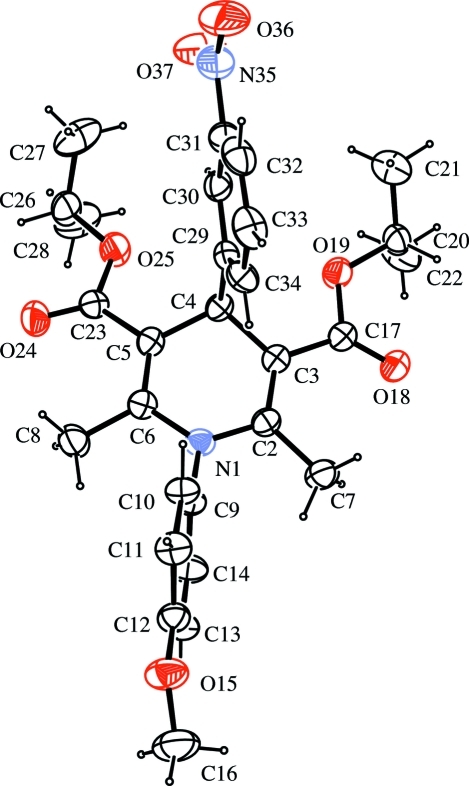
ORTEP view of the molecule with thedisplacement ellipsoids drawn at the 40% probability level. H atoms are shown as small spheres of arbitrary radii.

**Fig. 2.**
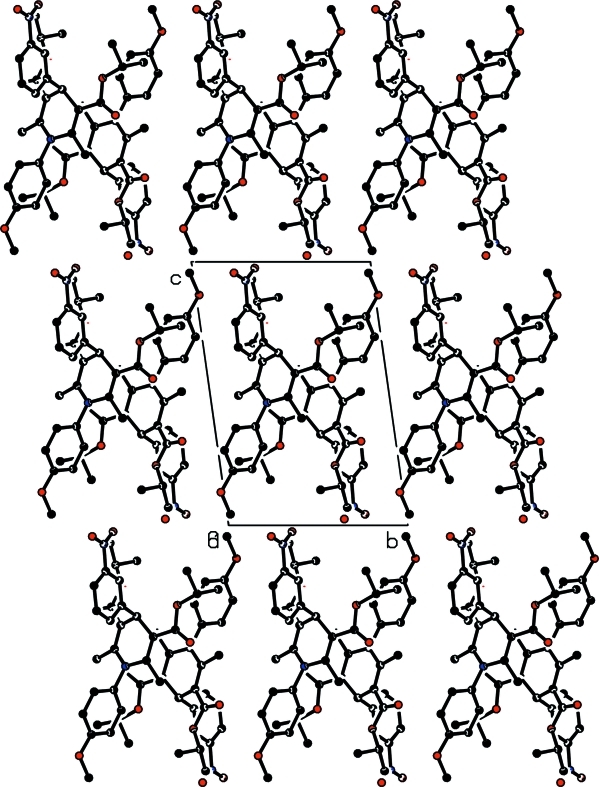
The packing arrangement of molecules viewed down the a-axis.

## supplementary crystallographic information

### Comment

Studies on 1,4-dihydropyridine (1,4-DHP) derivatives have been carried out in many research institutes all over the world because of their attractive biological activities ( Marchalin *et al.*, 2004; Chhillar *et al.*, 2006). 1,4-Dihydropyridines have played important role as chemotherapeutic agents, such as multi-drug resistance reversal in tumor cells (Shah *et al.*, 2000), potential immunomodulating (Shah *et al.*, 2002) and antitubercular compounds (Gaveriya *et al.*, 2001; Shah *et al.*, 2002). These compounds have also been investigated for other pharmacological activities such as antidiabetic, antiviral, antibacterial, membrane protecting, anticancer and antimicrobial. Calcium channel blockers of the 1,4-dihydropyridine derivatives, exemplified by nifedipine and nilvadipine, are well known as clinically important drugs since they first appeared on the market in 1975. To date, the structure-activity relationship of the DHPs has indicated that the desired structural characteristic of the substituents at the 4-position of the dihydropyridine nucleus had been thought to be the benzene ring. A favorable substituent on the 4-phenyl ring of DHP derivatives was suggested to be an electron-withdrawing group, such as the nitro group. Nitrophenyl substitution led to many cardiovascular drugs, namely, nilvadipine, nimodipine, nicardipine, nisoldipine, nifrendipine, *etc*. In view of the above, the crystal structure of the title compound was determined.

The classical preparation method of 1,4-DHP is the Hantzsch method. However, the classical methods were not enough to make pyridine libraries. Development of an efficient and versatile method for the preparation of 1,4 - dihydropyridines is an active ongoing research area and we have synthesized the title compound using catalytic method. In catalytic method the overall yields of the product are higher than the conventional classical method.

As in other dihydropyridine (DHP) structures, the DHP ring exhibits a flatened boat conformation. The N1 and C4 atoms lie 0.138 (3) and 0.336 (3) Å, respectively, from the least-squares plane defined by the remaining four atoms of the DHP ring. The puckering of the 1,4-DHP ring at N1 and C4, which is important for the biological activity of this class of compounds, is reflected in the torsion angles C3—C4—C5—C6 and C2—C3—C4—C5 which are 27.3 (4) and -26.5 (4)°o, respectively. The torsion angles about the bonds to N1 are -13.1 (5) (C2—N1—C6—C5) and 13.7 (5)° (C6—N1—C2—C3); All these values indicate that the puckering of the 1,4-DHP ring is largere at C4 site.

The values of the torsion angles, C6—N1—C9—C10 [-79.6 (4)°] and C5—C4—C29—C34 [79.9 (4)°]], describe the conformation around the inter-ring bond. The bezene rings are approximately perpendicular to the dihydropyridine ring. The dihedral angle found between the plane 1 (N1, C2, C3, C4, C5, C6) and plane 2 (C29, C30, C31, C32, C33, C34) is 84.29 (9)° and between the plane 1 (N1, C2, C3, C4, C5, C6) and plane 3 (C9, C10, C11, C12, C13, C14) is 82.96 (9)°. Owing to the absence of any strong donor group, cohesion of the crystal is mainly achieved by van der Waals interactions (Fig. 2)

### Experimental

A mixture of 3-nitrobenzaldehyde (5 mmol, 0.45 g), isopropyl acetoacetate (10 mmol, 1.44 g), 4-methoxyaniline (5 mmol, 0.615 g) was heated (without solvent) on steam bath for 2.5h. After elimination of water, iodine (1.5 mmol, 0.38 g) and ethanol (5 ml) were added to the reaction mixture.The reaction mixture was stirred, at room temperature, till the reaction was complet(4h monitored by TLC). The reaction mixture was treated with aqueous Na_2_S_2_O_3_ solution and the product was extracted with ethyl acetate (2*x* 20 ml). The solvent was removed under pressure and the resulting crude product (94%) was recrystallized from ethanol to give the analytical grade pure product. In catalytic method the overall yields of the product are higher than in the conventional classical method.

### Refinement

All H atoms were included in calculated positions and refined using a riding model approximation with C—H = 0.93–0.98 Å, and *U*_iso_(H) = 1.2U_eq_ (C), exept for the methyl groups where *U*_iso_(H) = 1.2U_eq_ (C).

### Crystal data

**Table:**

C_28_H_32_N_2_O_7_	*Z* = 2
*M**_r_* = 508.56	*F*(000) = 540
Triclinic, *P*1	*D*_x_ = 1.261 Mg m^−^^3^
Hall symbol: -P 1	Mo *K*α radiation, λ = 0.71073 Å
*a* = 9.5043 (8) Å	Cell parameters from 3153 reflections
*b* = 10.7570 (7) Å	θ = 3.4–29.0°
*c* = 15.1279 (12) Å	µ = 0.09 mm^−^^1^
α = 90.501 (6)°	*T* = 293 K
β = 105.873 (7)°	Block, light-yellow
γ = 114.601 (7)°	0.30 × 0.20 × 0.20 mm
*V* = 1339.27 (18) Å^3^	

### Data collection

**Table:**

Oxford Diffraction Xcalibur S diffractometer	4688 independent reflections
Radiation source: fine-focus sealed tube	2417 reflections with *I* > 2σ(*I*)
graphite	*R*_int_ = 0.043
Detector resolution: 16.1049 pixels mm^-1^	θ_max_ = 25.0°, θ_min_ = 3.4°
ω scans	*h* = −11→11
Absorption correction: multi-scan (*CrysAlis RED*; Oxford Diffraction, 2007)	*k* = −11→12
*T*_min_ = 0.825, *T*_max_ = 1.000	*l* = −17→17
8313 measured reflections	

### Refinement

**Table:**

Refinement on *F*^2^	Primary atom site location: structure-invariant direct methods
Least-squares matrix: full	Secondary atom site location: difference Fourier map
*R*[*F*^2^ > 2σ(*F*^2^)] = 0.069	Hydrogen site location: inferred from neighbouring sites
*wR*(*F*^2^) = 0.203	H-atom parameters constrained
*S* = 0.93	*w* = 1/[σ^2^(*F*_o_^2^) + (0.0993*P*)^2^] where *P* = (*F*_o_^2^ + 2*F*_c_^2^)/3
4688 reflections	(Δ/σ)_max_ = 0.001
334 parameters	Δρ_max_ = 0.23 e Å^−^^3^
0 restraints	Δρ_min_ = −0.23 e Å^−^^3^

### Special details

**Table:**

Experimental. *CrysAlis PRO*, Oxford Diffraction Ltd., Version 1.171.34.40 (release 27–08-2010 CrysAlis171. NET) (compiled Aug 27 2010,11:50:40) Empirical absorption correction using spherical harmonics, implemented in SCALE3 ABSPACK scaling algorithm.
Geometry. All e.s.d.'s (except the e.s.d. in the dihedral angle between two l.s. planes) are estimated using the full covariance matrix. The cell e.s.d.'s are taken into account individually in the estimation of e.s.d.'s in distances, angles and torsion angles; correlations between e.s.d.'s in cell parameters are only used when they are defined by crystal symmetry. An approximate (isotropic) treatment of cell e.s.d.'s is used for estimating e.s.d.'s involving l.s. planes.
Refinement. Refinement of *F*^2^ against ALL reflections. The weighted *R*-factor *wR* and goodness of fit *S* are based on *F*^2^, conventional *R*-factors *R* are based on *F*, with *F* set to zero for negative *F*^2^. The threshold expression of *F*^2^ > σ(*F*^2^) is used only for calculating *R*-factors(gt) *etc*. and is not relevant to the choice of reflections for refinement. *R*-factors based on *F*^2^ are statistically about twice as large as those based on *F*, and *R*- factors based on ALL data will be even larger.

### Fractional atomic coordinates and isotropic or equivalent isotropic displacement parameters (Å^2^)

**Table:**

	*x*	*y*	*z*	*U*_iso_*/*U*_eq_	
N1	0.5454 (3)	0.3122 (2)	0.46535 (16)	0.0543 (7)	
C2	0.6907 (4)	0.4335 (3)	0.4872 (2)	0.0506 (8)	
C3	0.7718 (4)	0.4905 (3)	0.5771 (2)	0.0488 (8)	
C4	0.7183 (3)	0.4147 (3)	0.6544 (2)	0.0473 (7)	
H4	0.7355	0.4837	0.7038	0.057*	
C5	0.5384 (4)	0.3176 (3)	0.6209 (2)	0.0476 (7)	
C6	0.4637 (4)	0.2640 (3)	0.5308 (2)	0.0505 (8)	
C7	0.7469 (4)	0.4920 (3)	0.4068 (2)	0.0689 (10)	
H7A	0.6708	0.4345	0.3500	0.103*	
H7B	0.8518	0.4952	0.4134	0.103*	
H7C	0.7541	0.5836	0.4054	0.103*	
C8	0.2886 (4)	0.1574 (4)	0.4926 (2)	0.0729 (10)	
H8A	0.2620	0.1341	0.4269	0.109*	
H8B	0.2187	0.1945	0.5048	0.109*	
H8C	0.2742	0.0761	0.5220	0.109*	
C9	0.4847 (4)	0.2280 (3)	0.3756 (2)	0.0514 (8)	
C10	0.5215 (4)	0.1169 (3)	0.3684 (2)	0.0547 (8)	
H10	0.5830	0.0971	0.4205	0.066*	
C11	0.4672 (4)	0.0366 (3)	0.2846 (2)	0.0628 (9)	
H11	0.4908	−0.0384	0.2800	0.075*	
C12	0.3773 (4)	0.0671 (3)	0.2067 (2)	0.0572 (8)	
C13	0.3389 (4)	0.1750 (3)	0.2140 (2)	0.0665 (9)	
H13	0.2775	0.1951	0.1619	0.080*	
C14	0.3912 (4)	0.2535 (4)	0.2984 (2)	0.0674 (10)	
H14	0.3627	0.3256	0.3033	0.081*	
O15	0.3339 (3)	−0.0171 (2)	0.12593 (16)	0.0828 (8)	
C16	0.2611 (6)	0.0187 (4)	0.0422 (3)	0.1070 (15)	
H16A	0.2367	−0.0487	−0.0085	0.161*	
H16B	0.3344	0.1079	0.0333	0.161*	
H16C	0.1626	0.0213	0.0448	0.161*	
C17	0.9226 (4)	0.6192 (3)	0.6040 (2)	0.0523 (8)	
O18	0.9944 (3)	0.6917 (2)	0.55631 (16)	0.0822 (8)	
O19	0.9781 (3)	0.6499 (2)	0.69719 (15)	0.0682 (7)	
C20	1.1316 (4)	0.7699 (3)	0.7403 (2)	0.0651 (9)	
H20	1.1983	0.7903	0.6983	0.078*	
C21	1.2148 (5)	0.7310 (5)	0.8269 (3)	0.1041 (14)	
H21A	1.2336	0.6538	0.8112	0.156*	
H21B	1.1475	0.7064	0.8670	0.156*	
H21C	1.3168	0.8079	0.8581	0.156*	
C22	1.0989 (5)	0.8909 (4)	0.7570 (4)	0.1150 (17)	
H22A	1.0482	0.9118	0.6987	0.172*	
H22B	1.1995	0.9693	0.7880	0.172*	
H22C	1.0281	0.8694	0.7950	0.172*	
C23	0.4521 (4)	0.2803 (3)	0.6907 (2)	0.0518 (8)	
O24	0.3176 (3)	0.1942 (3)	0.68094 (17)	0.0891 (9)	
O25	0.5433 (3)	0.3580 (3)	0.77256 (16)	0.0820 (8)	
C26	0.4739 (5)	0.3377 (4)	0.8499 (3)	0.0810 (12)	
H26	0.3717	0.2532	0.8336	0.097*	
C27	0.5956 (7)	0.3234 (5)	0.9321 (3)	0.1202 (17)	
H27A	0.6084	0.2420	0.9185	0.180*	
H27B	0.5577	0.3160	0.9854	0.180*	
H27C	0.6982	0.4030	0.9447	0.180*	
C28	0.4423 (6)	0.4586 (5)	0.8653 (3)	0.1123 (16)	
H28A	0.3603	0.4595	0.8119	0.169*	
H28B	0.5405	0.5418	0.8750	0.169*	
H28C	0.4055	0.4528	0.9189	0.169*	
C29	0.8208 (3)	0.3391 (3)	0.6953 (2)	0.0485 (8)	
C30	0.8803 (4)	0.3461 (3)	0.7905 (2)	0.0568 (8)	
H30	0.8585	0.3974	0.8303	0.068*	
C31	0.9715 (4)	0.2768 (4)	0.8259 (3)	0.0675 (9)	
C32	1.0054 (4)	0.2001 (4)	0.7710 (3)	0.0778 (11)	
H32	1.0644	0.1518	0.7971	0.093*	
C33	0.9512 (4)	0.1942 (3)	0.6758 (3)	0.0767 (11)	
H33	0.9768	0.1448	0.6371	0.092*	
C34	0.8584 (4)	0.2629 (3)	0.6389 (2)	0.0584 (9)	
H34	0.8204	0.2578	0.5748	0.070*	
N35	1.0345 (5)	0.2876 (4)	0.9286 (3)	0.0962 (11)	
O36	1.1211 (4)	0.2294 (4)	0.9581 (3)	0.1430 (14)	
O37	0.9971 (5)	0.3491 (4)	0.9761 (2)	0.1302 (13)	

### Atomic displacement parameters (Å^2^)

**Table:**

	*U*^11^	*U*^22^	*U*^33^	*U*^12^	*U*^13^	*U*^23^
N1	0.0572 (17)	0.0623 (16)	0.0429 (16)	0.0260 (15)	0.0145 (13)	0.0017 (13)
C2	0.056 (2)	0.0542 (18)	0.048 (2)	0.0272 (16)	0.0208 (17)	0.0066 (15)
C3	0.0532 (19)	0.0475 (16)	0.052 (2)	0.0259 (15)	0.0195 (16)	0.0089 (15)
C4	0.0547 (19)	0.0484 (16)	0.0477 (18)	0.0263 (15)	0.0224 (15)	0.0089 (14)
C5	0.0486 (18)	0.0518 (17)	0.054 (2)	0.0284 (15)	0.0228 (16)	0.0088 (15)
C6	0.0521 (19)	0.0541 (18)	0.052 (2)	0.0278 (16)	0.0183 (17)	0.0042 (15)
C7	0.080 (2)	0.076 (2)	0.052 (2)	0.033 (2)	0.0221 (19)	0.0109 (17)
C8	0.054 (2)	0.086 (2)	0.067 (2)	0.0203 (19)	0.0183 (19)	−0.0025 (19)
C9	0.0554 (19)	0.0587 (19)	0.0429 (19)	0.0289 (16)	0.0126 (16)	0.0034 (15)
C10	0.064 (2)	0.0585 (19)	0.0454 (19)	0.0339 (17)	0.0107 (16)	0.0081 (15)
C11	0.077 (2)	0.0546 (19)	0.061 (2)	0.0349 (19)	0.018 (2)	0.0045 (17)
C12	0.067 (2)	0.0543 (19)	0.047 (2)	0.0239 (17)	0.0171 (17)	0.0020 (16)
C13	0.072 (2)	0.071 (2)	0.052 (2)	0.035 (2)	0.0041 (18)	0.0044 (17)
C14	0.078 (2)	0.074 (2)	0.058 (2)	0.050 (2)	0.0054 (19)	0.0052 (18)
O15	0.112 (2)	0.0769 (16)	0.0501 (16)	0.0386 (15)	0.0150 (14)	−0.0045 (13)
C16	0.145 (4)	0.098 (3)	0.051 (3)	0.038 (3)	0.014 (3)	0.002 (2)
C17	0.057 (2)	0.0531 (18)	0.054 (2)	0.0285 (17)	0.0220 (18)	0.0110 (16)
O18	0.0751 (17)	0.0845 (17)	0.0591 (15)	0.0070 (14)	0.0230 (14)	0.0159 (13)
O19	0.0722 (16)	0.0624 (14)	0.0524 (15)	0.0109 (12)	0.0222 (12)	−0.0007 (11)
C20	0.056 (2)	0.063 (2)	0.060 (2)	0.0106 (18)	0.0172 (18)	0.0001 (17)
C21	0.086 (3)	0.120 (3)	0.085 (3)	0.037 (3)	0.006 (3)	0.008 (3)
C22	0.099 (3)	0.063 (2)	0.150 (5)	0.028 (2)	0.000 (3)	−0.014 (3)
C23	0.052 (2)	0.0596 (19)	0.053 (2)	0.0315 (17)	0.0172 (18)	0.0084 (17)
O24	0.0658 (17)	0.1032 (19)	0.0690 (17)	0.0041 (16)	0.0291 (14)	0.0024 (15)
O25	0.0675 (16)	0.1061 (19)	0.0551 (15)	0.0144 (14)	0.0309 (13)	−0.0085 (14)
C26	0.071 (2)	0.099 (3)	0.058 (2)	0.014 (2)	0.035 (2)	−0.006 (2)
C27	0.172 (5)	0.161 (5)	0.087 (3)	0.105 (4)	0.072 (4)	0.053 (3)
C28	0.152 (4)	0.158 (4)	0.080 (3)	0.103 (4)	0.058 (3)	0.027 (3)
C29	0.0398 (17)	0.0478 (17)	0.056 (2)	0.0168 (14)	0.0162 (16)	0.0078 (15)
C30	0.0509 (19)	0.062 (2)	0.064 (2)	0.0265 (17)	0.0235 (17)	0.0153 (17)
C31	0.057 (2)	0.070 (2)	0.074 (3)	0.029 (2)	0.015 (2)	0.0243 (19)
C32	0.053 (2)	0.067 (2)	0.115 (4)	0.033 (2)	0.015 (2)	0.022 (2)
C33	0.062 (2)	0.065 (2)	0.113 (4)	0.035 (2)	0.029 (2)	0.003 (2)
C34	0.0502 (19)	0.0586 (19)	0.064 (2)	0.0262 (17)	0.0107 (17)	−0.0004 (17)
N35	0.079 (3)	0.105 (3)	0.100 (3)	0.043 (2)	0.016 (2)	0.044 (2)
O36	0.127 (3)	0.195 (4)	0.128 (3)	0.100 (3)	0.019 (2)	0.077 (3)
O37	0.154 (3)	0.177 (4)	0.072 (2)	0.098 (3)	0.012 (2)	0.025 (2)

### Geometric parameters (Å, °)

**Table:**

N1—C6	1.395 (4)	O19—C20	1.455 (3)
N1—C2	1.401 (3)	C20—C22	1.492 (5)
N1—C9	1.453 (4)	C20—C21	1.495 (5)
C2—C3	1.356 (4)	C20—H20	0.9800
C2—C7	1.501 (4)	C21—H21A	0.9600
C3—C17	1.468 (4)	C21—H21B	0.9600
C3—C4	1.511 (4)	C21—H21C	0.9600
C4—C5	1.517 (4)	C22—H22A	0.9600
C4—C29	1.527 (4)	C22—H22B	0.9600
C4—H4	0.9800	C22—H22C	0.9600
C5—C6	1.346 (4)	C23—O24	1.193 (3)
C5—C23	1.468 (4)	C23—O25	1.332 (4)
C6—C8	1.514 (4)	O25—C26	1.470 (4)
C7—H7A	0.9600	C26—C28	1.481 (5)
C7—H7B	0.9600	C26—C27	1.507 (6)
C7—H7C	0.9600	C26—H26	0.9800
C8—H8A	0.9600	C27—H27A	0.9600
C8—H8B	0.9600	C27—H27B	0.9600
C8—H8C	0.9600	C27—H27C	0.9600
C9—C14	1.368 (4)	C28—H28A	0.9600
C9—C10	1.388 (4)	C28—H28B	0.9600
C10—C11	1.368 (4)	C28—H28C	0.9600
C10—H10	0.9300	C29—C30	1.385 (4)
C11—C12	1.384 (4)	C29—C34	1.389 (4)
C11—H11	0.9300	C30—C31	1.373 (5)
C12—C13	1.366 (4)	C30—H30	0.9300
C12—O15	1.370 (4)	C31—C32	1.353 (5)
C13—C14	1.370 (4)	C31—N35	1.488 (5)
C13—H13	0.9300	C32—C33	1.382 (5)
C14—H14	0.9300	C32—H32	0.9300
O15—C16	1.408 (5)	C33—C34	1.384 (5)
C16—H16A	0.9600	C33—H33	0.9300
C16—H16B	0.9600	C34—H34	0.9300
C16—H16C	0.9600	N35—O37	1.187 (4)
C17—O18	1.196 (3)	N35—O36	1.225 (4)
C17—O19	1.347 (4)		
			
C6—N1—C2	121.3 (2)	O19—C20—C22	109.1 (3)
C6—N1—C9	118.7 (2)	O19—C20—C21	106.6 (3)
C2—N1—C9	119.8 (2)	C22—C20—C21	113.8 (3)
C3—C2—N1	119.8 (3)	O19—C20—H20	109.1
C3—C2—C7	124.0 (3)	C22—C20—H20	109.1
N1—C2—C7	116.2 (3)	C21—C20—H20	109.1
C2—C3—C17	122.2 (3)	C20—C21—H21A	109.5
C2—C3—C4	120.3 (3)	C20—C21—H21B	109.5
C17—C3—C4	117.2 (3)	H21A—C21—H21B	109.5
C3—C4—C5	110.7 (2)	C20—C21—H21C	109.5
C3—C4—C29	111.3 (2)	H21A—C21—H21C	109.5
C5—C4—C29	111.7 (2)	H21B—C21—H21C	109.5
C3—C4—H4	107.6	C20—C22—H22A	109.5
C5—C4—H4	107.6	C20—C22—H22B	109.5
C29—C4—H4	107.6	H22A—C22—H22B	109.5
C6—C5—C23	121.8 (3)	C20—C22—H22C	109.5
C6—C5—C4	120.5 (3)	H22A—C22—H22C	109.5
C23—C5—C4	117.7 (3)	H22B—C22—H22C	109.5
C5—C6—N1	120.0 (3)	O24—C23—O25	120.5 (3)
C5—C6—C8	124.1 (3)	O24—C23—C5	128.0 (3)
N1—C6—C8	115.8 (3)	O25—C23—C5	111.5 (3)
C2—C7—H7A	109.5	C23—O25—C26	118.8 (3)
C2—C7—H7B	109.5	O25—C26—C28	107.1 (3)
H7A—C7—H7B	109.5	O25—C26—C27	106.9 (3)
C2—C7—H7C	109.5	C28—C26—C27	113.5 (3)
H7A—C7—H7C	109.5	O25—C26—H26	109.7
H7B—C7—H7C	109.5	C28—C26—H26	109.7
C6—C8—H8A	109.5	C27—C26—H26	109.7
C6—C8—H8B	109.5	C26—C27—H27A	109.5
H8A—C8—H8B	109.5	C26—C27—H27B	109.5
C6—C8—H8C	109.5	H27A—C27—H27B	109.5
H8A—C8—H8C	109.5	C26—C27—H27C	109.5
H8B—C8—H8C	109.5	H27A—C27—H27C	109.5
C14—C9—C10	119.0 (3)	H27B—C27—H27C	109.5
C14—C9—N1	122.2 (3)	C26—C28—H28A	109.5
C10—C9—N1	118.8 (3)	C26—C28—H28B	109.5
C11—C10—C9	120.1 (3)	H28A—C28—H28B	109.5
C11—C10—H10	120.0	C26—C28—H28C	109.5
C9—C10—H10	120.0	H28A—C28—H28C	109.5
C10—C11—C12	120.0 (3)	H28B—C28—H28C	109.5
C10—C11—H11	120.0	C30—C29—C34	118.1 (3)
C12—C11—H11	120.0	C30—C29—C4	120.5 (3)
C13—C12—O15	124.9 (3)	C34—C29—C4	121.4 (3)
C13—C12—C11	120.0 (3)	C31—C30—C29	119.6 (3)
O15—C12—C11	115.1 (3)	C31—C30—H30	120.2
C12—C13—C14	119.7 (3)	C29—C30—H30	120.2
C12—C13—H13	120.1	C32—C31—C30	122.4 (4)
C14—C13—H13	120.1	C32—C31—N35	119.5 (4)
C9—C14—C13	121.2 (3)	C30—C31—N35	118.1 (4)
C9—C14—H14	119.4	C31—C32—C33	119.2 (4)
C13—C14—H14	119.4	C31—C32—H32	120.4
C12—O15—C16	118.1 (3)	C33—C32—H32	120.4
O15—C16—H16A	109.5	C32—C33—C34	119.1 (3)
O15—C16—H16B	109.5	C32—C33—H33	120.5
H16A—C16—H16B	109.5	C34—C33—H33	120.5
O15—C16—H16C	109.5	C33—C34—C29	121.5 (3)
H16A—C16—H16C	109.5	C33—C34—H34	119.2
H16B—C16—H16C	109.5	C29—C34—H34	119.2
O18—C17—O19	121.0 (3)	O37—N35—O36	124.4 (5)
O18—C17—C3	129.6 (3)	O37—N35—C31	119.0 (4)
O19—C17—C3	109.4 (3)	O36—N35—C31	116.6 (4)
C17—O19—C20	119.2 (2)		
			
C6—N1—C2—C3	13.7 (4)	C12—C13—C14—C9	1.5 (6)
C9—N1—C2—C3	−161.0 (3)	C13—C12—O15—C16	7.9 (5)
C6—N1—C2—C7	−166.6 (3)	C11—C12—O15—C16	−171.8 (3)
C9—N1—C2—C7	18.7 (4)	C2—C3—C17—O18	0.8 (5)
N1—C2—C3—C17	−178.6 (2)	C4—C3—C17—O18	174.6 (3)
C7—C2—C3—C17	1.8 (5)	C2—C3—C17—O19	−178.8 (3)
N1—C2—C3—C4	7.8 (4)	C4—C3—C17—O19	−4.9 (3)
C7—C2—C3—C4	−171.9 (3)	O18—C17—O19—C20	−2.8 (4)
C2—C3—C4—C5	−26.5 (4)	C3—C17—O19—C20	176.8 (2)
C17—C3—C4—C5	159.5 (2)	C17—O19—C20—C22	94.3 (4)
C2—C3—C4—C29	98.4 (3)	C17—O19—C20—C21	−142.4 (3)
C17—C3—C4—C29	−75.6 (3)	C6—C5—C23—O24	7.0 (5)
C3—C4—C5—C6	27.3 (4)	C4—C5—C23—O24	−170.3 (3)
C29—C4—C5—C6	−97.4 (3)	C6—C5—C23—O25	−172.2 (3)
C3—C4—C5—C23	−155.4 (2)	C4—C5—C23—O25	10.5 (4)
C29—C4—C5—C23	80.0 (3)	O24—C23—O25—C26	−1.3 (5)
C23—C5—C6—N1	173.7 (3)	C5—C23—O25—C26	178.0 (3)
C4—C5—C6—N1	−9.0 (4)	C23—O25—C26—C28	−107.6 (4)
C23—C5—C6—C8	−2.6 (5)	C23—O25—C26—C27	130.4 (3)
C4—C5—C6—C8	174.7 (3)	C3—C4—C29—C30	134.9 (3)
C2—N1—C6—C5	−13.1 (4)	C5—C4—C29—C30	−100.8 (3)
C9—N1—C6—C5	161.7 (3)	C3—C4—C29—C34	−44.5 (4)
C2—N1—C6—C8	163.5 (3)	C5—C4—C29—C34	79.9 (3)
C9—N1—C6—C8	−21.7 (4)	C34—C29—C30—C31	−0.9 (4)
C6—N1—C9—C14	99.7 (4)	C4—C29—C30—C31	179.7 (3)
C2—N1—C9—C14	−85.5 (4)	C29—C30—C31—C32	−0.5 (5)
C6—N1—C9—C10	−79.6 (3)	C29—C30—C31—N35	179.5 (3)
C2—N1—C9—C10	95.3 (3)	C30—C31—C32—C33	2.2 (5)
C14—C9—C10—C11	1.3 (5)	N35—C31—C32—C33	−177.8 (3)
N1—C9—C10—C11	−179.4 (3)	C31—C32—C33—C34	−2.4 (5)
C9—C10—C11—C12	0.8 (5)	C32—C33—C34—C29	1.0 (5)
C10—C11—C12—C13	−1.8 (5)	C30—C29—C34—C33	0.6 (4)
C10—C11—C12—O15	177.9 (3)	C4—C29—C34—C33	−180.0 (3)
O15—C12—C13—C14	−179.0 (3)	C32—C31—N35—O37	−176.1 (4)
C11—C12—C13—C14	0.7 (5)	C30—C31—N35—O37	3.9 (6)
C10—C9—C14—C13	−2.5 (5)	C32—C31—N35—O36	2.9 (5)
N1—C9—C14—C13	178.3 (3)	C30—C31—N35—O36	−177.1 (3)
